# An analysis of health inequalities depending on educational level using nationally representative survey data in Japan, 2019

**DOI:** 10.1186/s12889-021-12368-2

**Published:** 2021-12-10

**Authors:** Tasuku Okui

**Affiliations:** Medical Information Center, Kyushu University Hospital, 3-1-1 Maidashi, Higashi-ku, Fukuoka, 812-8582 Japan

**Keywords:** Educational level, Japan, Health behavior, Health status disparities

## Abstract

**Background:**

In recent years, socioeconomic differences in health statuses and behaviors have not been investigated from the nationally representative survey data in Japan. In this study, we showed differences in representative health behaviors and statuses depending on educational level using a nationally representative survey data in Japan.

**Methods:**

Aggregated (not individual level) data from the Comprehensive Survey of Living Conditions in 2019 were used to examine the association between educational level and outcome status of psychological distress (K6 scores > = 5), self-rated health, smoking, alcohol drinking, and cancer screening participation (stomach, lung, colorectal, breast, and uterine cancers). Data of 217,179 households in Japan were aggregated by the Ministry of Health, Labour, and Welfare in the survey, and the data of the estimated number of household members and persons corresponding to each response option for the questions in all of Japan were used. Five-year age groups from 20 to 24 to 80–84 years and over 84 years were analyzed, and the prevalence or participation rate by educational level were calculated. In addition, the age-standardized prevalence or participation rate according to educational level were also calculated by sex. Moreover, a Poisson regression model was applied for evaluating an association of educational level with the outcomes.

**Results:**

As a result, a clear gradient by educational level was observed in almost all the age groups for the prevalence of psychological distress, poor self-rated health, and smoking and participation rates in cancer screening, and high educational level were associated with better health-related behaviors and statuses. Conversely, drinking prevalence was shown to be higher rather in highly educated people. In addition, a statistically significant association of educational level with all the outcomes was observed.

**Conclusion:**

It was shown that disparities in health behaviors and statuses still persisted in recent years, and the findings suggested that further measures should be taken to tackle this disparity.

## Background

Japan is famous for its high life expectancy, and the life expectancy is continuing to increase [1]. It is known that the age-standardized mortality rates of cancer and cardiovascular diseases, which are the largest causes of death in Japan, are continuing to decrease over recent decades [2]. However, it is known that socioeconomic disparities exist in cause-specific mortality rates or health behaviors and statuses in Japan [3–7]. It was demonstrated that socioeconomic disparities exist in mortality rates among municipalities for some causes of death in Japan in 2019 [7], and as a possible reason for the disparities, socioeconomic differences in health behaviors and statuses were pointed out [7].

Health inequality caused by differences in socioeconomic status is one of the major themes of public health in the world since a higher socioeconomic status is usually associated with a better health status [8–10]. Income, occupation, and educational level are often used as indicators of socioeconomic status [10–12], and health inequalities have been shown in all kinds of outcomes, such as life expectancies, smoking status, and prevalence of lifestyle-related diseases in the world [13–15]. In Japan, according to previous studies, the prevalence of smoking or psychological distress varied depending on income or educational levels [3, 16], and a higher income or educational level was shown to be associated with lower prevalence of smoking and psychological distress. In addition, it is known that the relationship between socioeconomic status and health behaviors or cause-specific mortality varied over the years in Japan [7, 16], and a possibility exists that the relationship between each type of health behavior or status changed over the years. However, socioeconomic differences in health indicators have not been revealed using the most recent nationally representative survey data. In previous studies [16–21], health inequalities by educational level in Japan have been shown using different data, research methods, and survey years. In addition, previous studies tend to focus on one health outcome, and a study focusing on multiple types of health outcomes have not been conducted in recent years. When we want to discuss health inequalities by educational level, it is better to investigate various kinds of health outcomes rather than one health outcome. By showing the differences in various kinds of health indicators by educational level using the most recent national data of Japan, the reality of health inequalities in the current time in Japan could be understood, and the most recent data on health-related outcomes in Japan could be also revealed.

The Comprehensive Survey of Living Conditions is a national survey conducted by the Ministry of Health, Labour and Welfare in Japan, and its data have been used for many previous studies for revealing the relationship between each type of health behavior or status and socioeconomic status [3, 4, 16, 20–22]. The most recent Comprehensive Survey of Living Conditions was conducted in 2019, whereas only one study analyzed the data [23]. The prevalence data of some types of health indicator depending on educational level are also publicly available, while the relationship in recent years has not been revealed yet using the most recent survey data. Therefore, in this study, we showed differences in representative health behavior or status depending on the educational level using aggregated data of the Comprehensive Survey of Living Conditions in Japan in 2019.

## Methods

### Data

**Fig. 1:**
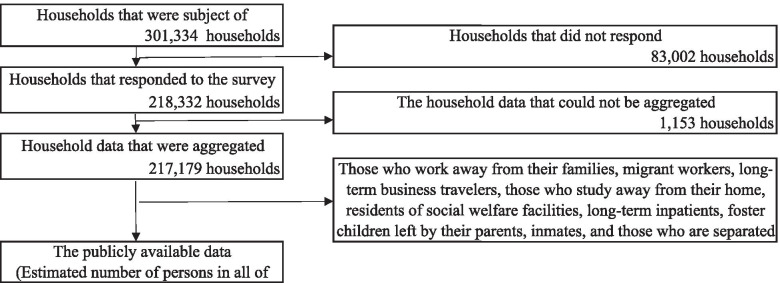
Flowchart of selecting study subjects in the survey

Aggregated Data (not individual level data) were derived from the Comprehensive Survey of Living Conditions in Japan for 2019 [24]. The survey is conducted by the Ministry of Health, Labour and Welfare on a yearly basis to obtain data on income status, savings, and households in Japan. In addition, health status is surveyed every 3 years. The subjects fill out self-reported questionnaires regarding their households. A total of 5530 districts (approximately 300,000 households) throughout Japan were selected through stratified random sampling, and all households in the selected districts (approximately 720,000 persons) were investigated in the survey [25]. The inclusion criteria are all persons living in households in selected districts are surveyed. Those who work away from their families, migrant workers, long-term business travelers, those who study away from their home, residents of social welfare facilities, long-term inpatients, foster children left by their parents, inmates, and those who are separated from their households were excluded in the survey [25]. In 2019, a total of 301,334 households became subjects for all forms of status, and the data of 218,332 households were gathered in the Ministry of Health, Labour, and Welfare [25]. The responses from 217,179 households were finally aggregated in the data after removing responses that cannot be aggregated [25]. The exact number of respondents for each question in the questionnaire was unknown. In addition, only those who were not hospitalized were included in the publicly available data. The estimated total number of persons in all of Japan corresponding to each response option of the questions in the survey was calculated using the prevalence of respondents for each response option and the Japanese population by the Ministry of Health, Labour, and Welfare, and this data are publicly available [24]. This study used the data on the status of psychological distress, self-rated health, smoking status, alcohol drinking frequency, and status of participation for cancer screening. The flowchart of selecting study subjects is shown in Fig. 1.

Data on educational level were provided as elementary school or junior high school, high school, vocational school, junior college or technical college, university, and graduate school. For the study, it was classified into three levels, namely, low (elementary school or junior high school), middle (high school and vocational school), and high (junior college or technical college, university, and graduate school) as it was done in a previous study [16]. Subjects with unknown educational level were not included in the analysis.

Psychological distress was assessed based on the scores of Kessler’s *Psychological* Distress Scale (K6), and subjects whose total score was ≥5 were classified as psychologically distressed. Regarding self-rated health, it was shown to be a valid indicator of overall health and an indicator of mortality in Japan [26]. In the survey, self-rated health was assessed by one question: “What is your current health status?” It was divided into two statuses, namely, good (very good, good, and normal) and poor (bad and very bad). Smoking status was classified into two, namely, smoker (i.e., smoking every day or sometimes) and non-smoker (i.e., former smoker and non-smoker). Regarding alcohol drinking frequency, the question was “How many days do you drink alcoholic beverages in a week?” The response options were “every day,” “5–6 days per week,” “3–4 days per week,” “1–2 days per week,” “1–3 days per month,” “merely drink,” “stop drinking,” and “not drinking.” Therefore, drinking status was classified into two, namely, drinker (i.e., drinking every day, 5–6 days per week, 3–4 days per week, 1–2 days per week, or 1–3 days per month) and non-drinker (i.e., merely drink, stop drinking, or not drinking).

Regarding cancer screening, the data of participation status for stomach, lung, colorectal, breast, and uterine cancer were available. According to the guideline for implementation of cancer screening in Japan [27], stomach cancer screening is recommended once every 2 years for persons aged ≥50 years. Lung and colorectal cancer screening is recommended once every year for persons aged ≥40 years. Breast cancer screening is recommended once every 2 years for persons aged ≥40 years, and uterine cancer screening is recommended once every 2 years for persons aged ≥20 years. Therefore, the participation status data with the recommended frequency for each cancer type were used. In addition, the data of patients aged ≥50 years for stomach cancer, aged ≥40 for lung, colorectal, and breast cancer, and ages of 25 or above for uterine cancer were used.

### Statistical analysis

Age groups in 5-year increments from 20 to 24 to 80–84 and the age group of over 84 years were available, and groups of 25–29 years or more were used because it is considered that many of the highly educated people had not graduated their university or graduate school yet by the age of 20–24 years. Therefore, the estimated number for each educational level does not contain those who are currently attending the corresponding educational level, and many people who are attending university or graduate school are not reflected in the data of the age group of 20–24. Prevalence of each health-related behaviors and statuses were calculated by age group, sex, and educational level.

In addition, age-standardized prevalence was calculated by sex and educational level using the sum of the estimated number of all the choices for each health-related behavior and status (estimated number of household persons in Japan) as the standard population. Specifically, we calculated number of household persons in Japan according to age group after removing number of persons whose responses (choices) for each outcome type were unknown. By multiplying the estimated number of household persons in Japan by the prevalence of outcomes for each age group and summing them by sex and educational level, we derived the expected number of persons with each health status or behavior in all of Japan for each sex and educational level. Then, by dividing the expected number of persons with each health status or behavior by the estimated total number of household persons in Japan, we derived the age-standardized prevalence of outcomes according to sex and educational level. Using this method, we were able to adjust for differences in age distribution depending on sex and educational level. We calculated age-standardized prevalence using the direct method previously described by Naing [28].

Moreover, Poisson regression analysis was conducted for evaluating an association of educational level with each health-related behavior and status using the data of each age group. In this analysis, educational level was used as factor variable (low educational level was used as reference), and each age group was adjusted as factor variable in the analysis. Lastly, statistical analysis was conducted using R3.6.3 [29].

## Results

Table 1 shows prevalence of each of the health-related behavior and status for each age group by educational level in men. The prevalence increased with an increase in educational level in all the age groups for psychiatric distress, poor self-rated health, and smoking status. Conversely, drinking prevalence became the highest in the high educational level in all the age groups. Participation rate in the three cancer screenings increased with an increase in educational level in all the age groups.

**Table 1:** Prevalence of health outcomes according to age and educational level in men

		Age groups												
**Health-related behaviors and statuses**	**Educational level**	**25–29**	**30–34**	**35–39**	**40–44**	**45–49**	**50–54**	**55–59**	**60–64**	**65–69**	**70–74**	**75–79**	**80–84**	**over 84**
Prevalence of psychological distress^a^	Total	30.5	33.1	33.3	30.3	29.5	28.1	28.4	26.1	22.1	20.1	18.6	22.2	25.5
	Low	40.4	31.8	32.4	30.6	29.4	29.3	29.7	30.8	27.4	23.8	22.6	24.9	28.0
	Middle	30.0	32.5	32.0	29.9	30.0	28.7	28.1	27.4	23.5	20.2	19.2	23.2	25.1
	High	30.2	33.7	34.7	30.7	29.0	27.0	28.5	24.3	19.9	18.5	15.4	17.1	22.4
Prevalence of poor self-rated health	Total	5.3	6.5	8.1	8.0	8.2	9.3	10.6	11.8	12.3	14.4	17.4	21.9	29.7
	Low	8.8	10.4	11.6	12.5	13.0	12.2	12.8	20.0	18.7	18.0	22.5	24.4	30.1
	Middle	5.6	6.5	7.5	8.3	8.1	10.1	11.1	12.7	13.4	15.1	17.0	22.2	29.5
	High	4.4	6.1	8.2	7.3	7.7	8.1	9.8	9.9	10.3	12.1	14.8	18.0	29.8
Smoking prevalence	Total	31.8	34.7	36.3	37.4	36.9	35.7	33.7	30.8	26.4	20.3	13.8	8.6	6.5
	Low	59.7	66.4	54.7	54.6	58.7	53.7	51.0	46.9	35.2	25.3	15.2	11.4	7.3
	Middle	38.9	42.0	45.3	45.3	42.8	42.6	41.0	35.5	29.1	21.3	14.4	8.6	6.6
	High	23.0	24.9	26.0	27.1	26.7	25.1	23.4	23.5	19.2	15.5	10.7	4.1	4.9
Drinking prevalence	Total	48.6	53.2	56.1	59.6	60.7	63.9	66.9	66.8	63.3	58.9	54.8	46.7	36.7
	Low	47.1	51.8	49.3	53.0	55.4	54.8	57.3	60.7	59.4	52.5	50.3	40.8	33.1
	Middle	42.1	48.1	52.1	56.5	58.1	63.2	65.7	67.0	62.9	59.1	54.9	49.5	37.2
	High	54.7	58.1	60.5	63.7	64.7	66.0	69.4	67.6	65.4	62.4	59.9	50.1	42.6
Participation rate in stomach cancer screening^b^	Total						57.9	60.9	58.1	51.2	49.1	47.5	42.5	31.1
	Low						32.4	35.6	36.1	36.2	39.0	39.3	37.1	27.1
	Middle						51.1	54.8	53.6	50.3	49.1	48.8	43.8	32.6
	High						69.5	70.6	66.1	58.3	55.5	54.6	48.3	36.0
Participation rate in lung cancer screening^c^	Total				53.4	56.0	57.7	62.3	58.3	51.8	50.5	46.5	41.9	35.2
	Low				35.9	35.8	36.9	39.6	38.8	40.1	41.2	41.5	39.0	34.1
	Middle				48.3	52.3	53.3	57.0	55.7	51.6	50.7	47.3	43.1	34.5
	High				60.8	63.1	65.8	70.7	64.1	56.6	56.0	51.0	44.1	38.8
Participation rate in colorectal cancer screening^c^	Total				47.3	49.6	51.6	55.9	52.0	47.0	46.8	43.7	38.7	28.8
	Low				25.3	27.7	30.9	31.0	32.6	32.9	37.4	36.5	33.3	24.2
	Middle				41.6	45.0	46.3	50.3	48.8	46.2	46.2	44.8	40.0	28.6
	High				56.0	58.1	60.7	64.8	58.3	53.5	53.6	50.3	44.2	38.1

^a^Proportion of persons with K6 scores ≥5

^b^Only data for individuals aged 50 years and over are shown considering the target screening age

^c^Only data for individuals aged 40 years and over are shown considering the target screening age

Table 2 shows prevalence of each of the health-related behavior and status for each age group by educational level in women. Results were relatively similar to those of men. The peak prevalence of psychiatric distress was in the fourth decade for men, and the prevalence tended to decrease in older ages. In contrast, a trend toward decreasing prevalence with older age was less evident for women.

**Table 2:** Prevalence of health outcomes according to age and educational level in women

		Age groups												
**Health-related behaviors and statuses**	**Educational level**	**25–29**	**30–34**	**35–39**	**40–44**	**45–49**	**50–54**	**55–59**	**60–64**	**65–69**	**70–74**	**75–79**	**80–84**	**over 84**
Prevalence of psychological distress^a^	Total	34.8	37.7	36.0	34.1	33.5	34.9	34.8	32.8	27.6	23.9	23.7	26.1	32.3
	Low	37.2	43.5	47.3	50.5	45.1	51.3	39.8	36.5	31.2	26.2	27.4	30.3	34.3
	Middle	34.5	37.4	37.8	35.8	35.0	36.1	36.1	33.8	28.1	24.2	23.3	24.6	31.2
	High	35.1	37.5	33.5	31.2	31.5	32.3	32.4	31.0	26.2	21.7	20.5	20.7	28.0
Prevalence of poor self-rated health	Total	7.0	9.0	10.6	9.9	10.9	12.6	12.8	12.8	12.5	13.6	16.9	23.0	30.5
	Low	13.0	11.1	18.1	20.2	18.1	27.5	23.5	22.2	17.2	18.1	21.1	25.6	30.6
	Middle	7.1	9.8	10.1	10.8	12.1	13.3	13.7	13.5	13.6	13.9	16.5	22.0	31.1
	High	6.3	8.1	10.4	8.3	9.3	10.6	10.5	10.9	9.7	9.9	12.8	19.8	26.2
Smoking prevalence	Total	9.5	10.7	10.9	12.1	13.4	11.8	11.0	9.4	7.0	5.4	3.0	1.8	1.2
	Low	32.8	40.4	42.2	42.6	45.0	35.0	26.3	17.3	10.8	7.9	4.4	2.1	1.1
	Middle	13.9	15.2	15.0	17.3	18.2	15.4	14.0	10.7	7.3	5.0	2.5	1.8	1.3
	High	4.1	4.0	4.4	5.4	5.1	4.2	4.9	6.0	4.2	3.5	1.0	0.6	0.9
Drinking prevalence	Total	33.0	30.7	33.9	38.4	40.0	38.5	36.8	33.3	26.9	22.2	17.2	12.6	8.2
	Low	28.6	36.8	33.0	34.7	40.0	36.6	30.6	24.2	21.5	17.7	16.1	11.2	6.8
	Middle	29.2	31.2	34.3	37.8	38.6	37.7	35.3	32.5	26.8	22.8	17.4	13.4	9.2
	High	36.6	29.7	33.5	39.1	41.7	39.9	39.6	36.2	30.2	25.7	19.7	15.3	14.2
Participation rate in stomach cancer screening^b^	Total						48.3	49.2	46.1	44.3	43.2	41.5	34.1	19.6
	Low						26.3	35.1	29.1	36.4	36.4	36.7	31.3	17.8
	Middle						44.3	45.9	44.0	44.2	44.1	43.3	35.8	20.7
	High						56.5	55.7	52.5	48.9	48.3	46.6	40.1	27.9
Participation rate in lung cancer screening^c^	Total				44.2	47.3	50.9	51.8	47.3	45.6	44.6	41.2	34.8	24.4
	Low				25.8	30.5	28.6	36.5	32.8	39.4	38.0	37.6	32.5	23.4
	Middle				39.8	43.8	48.0	49.6	46.2	46.0	45.9	43.0	35.7	25.1
	High				49.3	52.9	57.4	56.5	51.6	48.0	47.7	42.5	42.8	29.1
Participation rate in colorectal cancer screening^c^	Total				39.2	42.2	45.0	45.9	42.6	41.3	41.1	38.4	30.3	16.6
	Low				23.7	25.4	24.5	27.1	27.8	33.1	34.7	33.1	26.2	14.6
	Middle				34.4	37.9	41.7	42.9	41.1	41.1	42.1	40.5	32.9	18.1
	High				44.7	48.8	52.0	52.1	47.5	46.3	45.5	43.5	36.9	22.3
Participation rate in breast cancer screening^c^	Total				55.6	54.7	54.4	52.0	44.5	37.8	31.5	23.2	13.9	5.6
	Low				28.3	30.8	27.8	28.6	23.9	31.5	23.4	17.6	10.9	4.0
	Middle				49.1	49.0	50.4	47.9	42.1	37.0	32.3	25.0	15.7	6.9
	High				63.3	63.7	62.9	60.4	51.9	43.5	38.8	30.7	20.0	10.0
Participation rate in uterus cancer screening	Total	38.4	51.2	55.4	58.2	55.0	53.0	46.9	38.5	32.3	26.1	18.8	11.4	4.6
	Low	32.3	35.5	37.5	34.4	36.8	27.6	26.8	21.0	26.1	20.3	15.6	9.2	4.1
	Middle	34.6	47.9	49.9	52.3	49.6	49.6	42.4	36.4	32.0	26.8	19.8	12.4	5.1
	High	42.1	55.7	62.0	65.2	63.0	60.5	55.6	44.8	36.3	30.8	22.7	17.5	5.5

^a^Proportion of persons with K6 scores ≥5

^b^Only data for individuals aged 50 years and over are shown considering the target screening age

^c^Only data for individuals aged 40 years and over are shown considering the target screening age

Table 3 shows the age-standardized prevalence of each of the health-related behaviors and status by educational level among men and women. The age-standardized prevalence or participation rate ameliorated with an increase in educational level for all the health-related behaviors and statuses except drinking status.

**Table 3:** Age-standardized prevalence of each health outcome according to educational level among men and women

Health-related behaviors and statuses, sex, and educational level	Men^b^				Women^b^			
	Total	Low	Middle	High	Total	Low	Middle	High
Prevalence of psychological distress^a^	26.1	28.6	26.4	24.8	31.2	38.3	31.7	28.7
Prevalence of poor self-rated health	12.6	16.7	13.0	11.2	14.0	21.0	14.4	11.6
Smoking prevalence	28.5	43.1	33.4	20.6	8.8	24.8	11.2	4.0
Drinking prevalence	58.4	52.7	57.0	61.9	30.0	27.1	29.6	32.2
Participation rate in stomach cancer screening	51.6	35.7	49.3	59.5	42.7	32.0	41.9	48.9
Participation rate in lung cancer screening	52.8	38.4	50.6	58.9	44.7	33.0	43.6	49.2
Participation rate in colorectal cancer screening	47.5	31.3	44.8	55.1	39.9	27.7	38.5	45.6
Participation rate in breast cancer screening					39.9	24.2	37.8	47.3
Participation rate in uterus cancer screening					39.2	25.8	36.8	45.0

^a^Proportion of persons whose score of K6 > =5

^b^Age-specific estimated number of total educational levels was used as standard population for each health outcome

Table 4 shows results of the Poisson regression analysis evaluating an association of educational level with the health-related behaviors and status among men and women. Statistically significant associations of the educational level were observed for all the health-related behaviors and statuses.

**Table 4:** Poisson regression analysis evaluating the association between educational level and health outcomes

	Men		Women	
	Middle level vs. Low level^b^	High level vs. Low level^c^	Middle level vs. Low level^b^	High level vs. Low level^c^
**Health-related behaviors and statuses**	**PR (95% CI)**^a^	**PR (95% CI)**^a^	**PR (95% CI)**^a^	**PR (95% CI)**^a^
Prevalence of psychological distress^d^	0.89 (0.83, 0.96)	0.85 (0.79, 0.91)	0.85 (0.79, 0.90)	0.77 (0.72, 0.83)
Prevalence of poor self-rated health	0.83 (0.76, 0.90)	0.71 (0.65, 0.78)	0.82 (0.76, 0.88)	0.64 (0.58, 0.70)
Smoking prevalence	0.80 (0.76, 0.86)	0.49 (0.46, 0.53)	0.51 (0.46, 0.57)	0.17 (0.15, 0.19)
Drinking prevalence	1.09 (1.04, 1.15)	1.19 (1.13, 1.25)	1.17 (1.08, 1.26)	1.25 (1.16, 1.36)
Participation rate in stomach cancer screening	1.28 (1.21, 1.37)	1.57 (1.47, 1.68)	1.21 (1.13, 1.28)	1.43 (1.33, 1.53)
Participation rate in lung cancer screening	1.22 (1.15, 1.29)	1.44 (1.35, 1.52)	1.18 (1.11, 1.25)	1.36 (1.27, 1.44)
Participation rate in colorectal cancer screening	1.32 (1.24, 1.40)	1.63 (1.53, 1.74)	1.26 (1.19, 1.34)	1.52 (1.42, 1.63)
Participation rate in breast cancer screening			1.43 (1.33, 1.55)	1.80 (1.67, 1.95)
Participation rate in uterus cancer screening			1.34 (1.25, 1.45)	1.65 (1.53, 1.79)

*PR* Prevalence ratio (participation rate ratio), *CI* Confidence interval

^a^The effect of age was adjusted for in the regression analysis

^b^PR of middle educational level compared with low educational level

^c^PR of high educational level compared with low educational level

^d^Proportion of persons with K6 scores ≥5

## Discussion

This study revealed educational-level-dependent differences in health-related behaviors and statuses in Japan. This study revealed prevalence of health outcomes by sex, educational level, and age group using the most recent data in Japan using the most recent national data for the first time. As the results revealed, disparities depending on the educational level existed in most of the behaviors and statuses. We discuss the disparity in each of the health-related behaviors and statuses.

Regarding psychological distress, it was shown that people with low educational level tended to be psychologically distressed. Employment status and income are known to be highly associated with psychological distress [3, 30], and it is considered that educational level is associated with psychological distress through employment status. It is said that the association among psychological outcomes, incomes, and employment status are interactive [3], and it is considered that low socioeconomic status leads to psychological distress. In turn, low socioeconomic status results in psychological distress. In addition, the possibility exists that educational differences are present in stress-coping skills.

Regarding self-rated poor health, results were relatively similar to psychological distress. Although it was known that the degree of the disparity in self-rated health depending on educational level was relatively low in Japan [22], disparities were observed in all the age groups for both genders in this study. Given that the previous study was based on the data in 2013 [22], a possibility exists that the tendency changed over the years. Health literacy is shown to be one factor affecting the self-rated health of Japanese people [31], and it possibly mediates the association between self-rated health and educational level.

Regarding smoking prevalence, it is known that the disparities depending on educational level increased from 2010 to 2016 in Japan [16]. As the results of this study showed, the degree of the disparities is large, particularly in younger aged persons. It was shown that smoking prevalence decreases in older ages also in other countries [32, 33], and as an explanation for the disparities in the younger ages, it is said that educational level is associated with the social class, particularly in younger people [20]. Also, it is pointed out that the opportunities to quit smoking are fewer for less educated people [20]. It is known that low socioeconomic status people who demonstrate a lower ability to process health-related information exhibit lesser social support [34, 35], and these things might have affected the disparity.

Regarding the drinking prevalence, the prevalence was rather higher in highly educated people. Risky alcohol behavior is often shown to be associated with lower educational level [36–38], whereas alcohol drinking habits are not necessarily positively associated with lower educational level [38]. In Japan, it is known that drinking habits are rather prevalent in people with higher socioeconomic statuses in adulthoods [38], whereas drinking habits in high school students was shown to be associated with lower educational level of their parents [39]. Alcohol drinking is considered as a method of social interaction in adulthood, and the prevalence was shown to be high in high income earners or employed persons in the previous study [38]. It is pointed out that enough money to purchase alcoholic beverages and work-related networking can explain the association between higher income and alcohol consumption [38]. A study in England also showed that those in the two lowest occupational categories had fewer drinking occasions than those with professional-managerial occupations [40]. A similar association between higher income and drinking frequency was observed in New Zealand [41]. Therefore, more alcohol drinking occasions in higher occupational classes could lead to the association between higher drinking prevalence and higher educational level in Japan. Conversely, a low socioeconomic status was shown to be associated with problematic heavy alcohol consumption [38], and it is considered that highly educated people are capable of consuming alcohol moderately.

Regarding participation rates in cancer screening, the disparity depending on educational level existed in all types of cancer screening. Some possible explanations exist for this phenomenon. First, Japanese people mainly participate in cancer screening conducted by municipalities or workplaces. Unemployed or self-employed persons exhibit less chance to participate in cancer screening conducted by workplaces, and their participation rates were shown to be lower than those of people who are working in companies [4, 19]. It is considered that educational level and employment type or status are related, and the relationship affected the results. In addition, charges are generally incurred when participating in cancer screening conducted by municipalities, possibly contributing to the disparity. Moreover, a possibility exists that knowledge about cancer screening or motivation for participation in cancer screening vary depending on the level of education [17, 19]. It is known that socioeconomic status is associated with health literacy [31, 42], and it possibly contributed to the lower participation rates.

The results of this study showed that educational differences in many types of health-related behaviors and statuses persisted even in recent years. Although it is not certain whether educational level is the fundamental cause of the disparity in these behaviors and statuses, it is considered that some common causes that are at least associated with educational level exist for these behaviors and statuses. Health literacy is a major factor associating unhealthy behaviors with low socioeconomic status, and individuals with low educational background tend to display low levels of health literacy (i.e., lack of knowledge about health behavior, inability, or indifference to understand the benefit of health behaviors) [31]. A method should be formulated for improving health literacy particularly for less educated individuals. In addition, it is considered that lifestyles of individuals are affected by their environments, and behaviors of people with low educational levels are affected by those of other less educated people. Although it is not easy to solve this problem, education of health-related behaviors or health literacy needs to be introduced more from childhood in order to ease the disparities depending on educational levels. Furthermore, regarding cancer screening, making participation in cancer screening free of charge is one method of easing the disparity depending on the socioeconomic status. Recently, municipalities often distribute a coupon ticket for some types of cancer screening in Japan, and these attempts are considered to be one method for easing the disparity.

### Limitations

As a limitation of this study, the survey data are based on self-reported questionnaires, which might have led to certain inaccurate responses. Moreover, we could obtain only data on the frequency for drinking, and we could not obtain data on the amount of drinking. By combining the data on the frequency and amount of drinking, we could evaluate the prevalence of heavy alcohol drinking. Furthermore, only the estimated number in Japan for each type of behavior and status can be obtained from the aggregated data and individuals’ data cannot be obtained at this point for the 2019 data. Therefore, exact number of respondent of each choice of the questions and number of persons with each of the educational level surveyed is unknown. If individuals’ data could be analyzed, we could take into account other characteristics of individuals in the analysis. Therefore, an analysis using individuals’ data is also warranted in the future.

## Conclusion

We revealed the relationship between educational level and some health-related behaviors and statuses using the aggregated data of the Comprehensive Survey of Living Conditions in 2019. As a result, a clear gradient by educational level was observed in almost all the age groups for the prevalence of psychological distress, poor self-rated health; smoking and participation rates in cancer screening and high educational level were associated with better health-related behaviors and statuses. Conversely, drinking prevalence was shown to be higher rather in highly educated people. It was shown that disparities in health-related behaviors and statuses still persisted in recent years, and the results suggested that further measures should be taken to tackle this disparity.

## Data Availability

The data used in this study can be obtained from websites of government statistics in Japan (Comprehensive Survey of Living Conditions. [cited 2021 31 March]. Available from: https://www.e-stat.go.jp/stat-search/files?page=1&toukei=00450061).
